# The Immunomodulatory Effects of Mesenchymal Stem Cells on Regulatory B Cells

**DOI:** 10.3389/fimmu.2020.01843

**Published:** 2020-08-14

**Authors:** Jialing Liu, Qiuli Liu, Xiaoyong Chen

**Affiliations:** ^1^The Biotherapy Center, The Third Affiliated Hospital of Sun Yat-sen University, Guangzhou, China; ^2^Center for Stem Cell Biology and Tissue Engineering, Key Laboratory for Stem Cells and Tissue Engineering, Ministry of Education, Sun Yat-sen University, Guangzhou, China; ^3^Department of Pathophysiology, Zhongshan School of Medicine, Sun Yat-sen University, Guangzhou, China

**Keywords:** mesenchymal stem cells (MSCs), regulatory B cells (Bregs), cell-to-cell contact, soluble factors, extracellular vesicles (EVs)

## Abstract

The therapeutic potential of mesenchymal stem cells (MSCs) has been investigated in many preclinical and clinical studies. This potential is dominantly based on the immunosuppressive properties of MSCs. Although the therapeutic profiles of MSC transplantation are still not fully characterized, accumulating evidence has revealed that B cells change after MSC infusion, in particular inducing regulatory B cells (Bregs). The immunosuppressive effects of Bregs have been demonstrated, and these cells are being evaluated as new targets for the treatment of inflammatory diseases. MSCs are capable of educating B cells and inducing regulatory B cell production via cell-to-cell contact, soluble factors, and extracellular vesicles (EVs). These cells thus have the potential to complement each other's immunomodulatory functions, and a combined approach may enable synergistic effects for the treatment of immunological diseases. However, compared with investigations regarding other immune cells, investigations into how MSCs specifically regulate Bregs have been superficial and insufficient. In this review, we discuss the current findings related to the immunomodulatory effects of MSCs on regulatory B cells and provide optimal strategies for applications in immune-related disease treatments.

## Introduction

Mesenchymal stem cells (MSCs) are multipotent stromal cells existing in many human tissues that can be rapidly expanded *in vitro* to meet the needs of clinical and basic research. The term MSCs was coined by Caplan in 1991 (1). Since Friedenstein and coworkers demonstrated the osteogenic potential of a minor subpopulation of BM cells that rapid adherence to tissue culture vessels and have a fibroblast-like appearance of their progeny in culture (2), MSCs have been derived from lots of tissues in different species (3, 4). However, MSCs still lack specific markers for identification. The International Society for Cell Therapy (ISCT) established three basic criteria for the identification of MSCs in 2006: (1) demonstration of plastic-adherent growth; (2) exhibition of the following phenotypic characteristics: expression of CD105, CD73, and CD90 in more than 95% of cells; a lack of expression of CD45, CD34, CD14, CD11b, CD79a, and CD19 in the majority of cells; and a lack of expression of HLA-DR; and (3) demonstration of the ability to differentiate into osteoblasts, adipocytes, chondroblasts *in vitro* (5). MSCs can exhibit important roles in tissue regeneration and repair (6), maintenance of bone marrow hematopoietic microenvironment homeostasis (7), and immunomodulation of inflammation (8).

Given the current considerable safety and efficacy in pre-clinical and clinical studies, the roles of MSCs in regenerative medicine have attracted widespread attention, especially their immunomodulatory effects on autoimmune diseases and transplantions, such as Crohn's disease (CD) (9), rheumatoid arthritis (RA) (10), and systemic lupus erythematosus (SLE) (11), as well as graft-versus-host disease (GvHD) (12), kidney transplantation (KTx) (13, 14), liver transplantation (LTx) (15, 16), chronic lung allograft dysfunction (CLAD) (17) and small bowel transplantation (SBTx) (18), and even their roles in immune-mediated cell therapies (19). MSCs exhibit functional characteristics related to immune regulation and have consistently been shown to play roles in regulating innate and adaptive immune responses via a variety of pathways, such as cell-to-cell contact (20), soluble factors (21), and exosomes derived from MSCs (22). For instance, MSCs possess the ability to secrete regulatory molecules and cytokines that can modulate PBMC maturation, proliferation, differentiation, migration, and functional activation (23–25).

B cells are essential immune effector cells that are pivotal in adaptive immune responses and play roles in autoimmunity through antigen presentation, antibody secretion, and complement activation. Previous studies have shown that MSCs are capable of regulating B cell proliferation and differentiation, inhibiting B cell apoptosis, etc., and they can also suppress the adaptive immune response by indirectly regulating dendritic cell (DC)-mediated antigens. Another mechanism by which MSCs may exert effects on autoimmune diseases in the short and long term is their induction of regulatory B cells (Bregs), especially types that promote the secretion of interleukin (IL)-10, which promote B cells to exhibit immunosuppressive functions and modulate the immune environment homeostasis of patients with autoimmune diseases or solid organ transplantation such kidney transplantation and liver transplantation.

A relatively large number of studies have been published to confirm the clinical phenomenon and mechanisms regarding MSCs regulating regulatory B cells. In addition, previous studies have shown the regulatory effects in animal disease models and the safety, feasibility and potential effectiveness of allogeneic transplantation of MSCs in clinical trials to treat immune-related diseases. It seems necessary to better understand how the underlying mechanisms of MSC-mediated Breg or combined MSC/Breg cell therapies can be successfully applied in clinical fields. In this review, we discuss MSC functions related to Bregs and the possible mechanisms by which MSCs induce Bregs *in vivo* and *in vitro*, especially with regard to IL-10-producing Bregs.

## Current Definition and Understanding of Regulatory B Cells

B cells, an important cells for the adaptive immune response, have the ability to present antigens, secrete antibodies, and activate the immune system (26), which have been observed in autoimmune diseases, infections and cancers. Several subsets of B cells exert regulatory functions similar to those of regulatory T cells (Tregs) and are collectively termed regulatory B cells (Bregs). Previous studies have shown that Bregs could inhibit Th1 and Th17 responses and induce ${\text{FoxP}}_{3}^{+}$ Treg pools to play a key role in maintaining peripheral tolerance (27). Regulatory B cells have been found in various B cell subpopulations, including B1 B cells, B2 B cells, and plasma cells (28). Breg-mediated immunosuppression is an important manner for the maintenance of peripheral tolerance (29). However, there is still no clear consensus on the definition and classification of Bregs. As their heterogeneity, Bregs may express one or more of regulatory factors [including IL-10, IL-35, transforming growth factor (TGF)-β, and programmed cell death 1 ligand 1 (PD-L1)] and exert suppressive effects on cognate T cells (27, 30–32). Since three inhibitory cytokines, IL-10, TGF-β, and IL-35, having been identified as key inhibitory inflammatory factors for Bregs, Bregs can be divided into three categories: IL-10^+^, TGF-β^+^, and IL-35^+^ Bregs. Among these, the IL-10^+^ Bregs, also called B10 cells, are the major cell type in mediating immunosuppression. IL-10^+^ Bregs have been widely regarded as important immunoregulatory cells in various inflammatory diseases, such as RA (33), chronic intestinal inflammatory conditions (34), SLE (35), CD (36), Collagen Induced Arthritis (CIA) (37), and GVHD (38). Besides, Bregs also play an important role in transplantation, including KTx (39, 40), cardiac allografts (41), liver transplantation (42) and so on. The various subpopulation phenotypes among IL-10^+^ Bregs are shown in Table 1.

**Table 1:** Phenotypes of IL-10^+^ Bregs.

**Species**	**Phenotype**	**Function**
Mouse	CD138^high^ (43)	Anti-Salmonella immunity
	CD19^+^CD5^+^CD1d^high^ (44, 45)	Treg induction; inhibition of Th17 response
	CD1d^high^CD23^high^CD21^int^ (46)	Protective role in the mucosa
	CD19^+^CD43^+^CD80^+^CD86^+^CD40^+^ (47)	Inhibition of Th1 response
	CD19^+^CD43^+^CD5^+^ (48)	Amelioration of cGVHD
	CD1d^high^ (49)	Treg induction
	CD5^+^CD1d^hi^ (41)	Inhibition of Th1 cells activation; induction of islet allograft tolerance
	CD19^+^CD24^high^CD38^high^ (30)	Suppression of Th1 cell differentiation
	IL-21R^+^ MZP (50)	Induction transplantation tolerance
Human	CD5^+^IL-10^+^ (51)	Inhibition of Th1 response
	CD19^+^CD25^high^CD27^high^CD86^high^CD1d^high^IL-10^high^TGF-β^high^ (52)	Suppression of CD4+ T cell proliferation
	CD19^+^CD38^+^CD1d^+^IgM^+^CD147^+^CD25^+^ (53)	Suppression of antitumor immune responses
	CD24^high^CD27^+^ (42, 54)	Negatively regulate monocyte cytokine production; predicted the occurrence of acute allograft rejection in liver transplantation
	CD154^+^ (55)	A character of SLE patients
	CD25^+^CD71^+^CD73^low^PD-L1^+^ (56)	Suppress antigen-specific immune responses
	CD27^int^CD38^+^ (57)	Production of IL-10
	CD5^high^CD38^low^PD-1^high^ (58)	Inhibition of Th1 and Th17 differentiation
	CD23^+^CD43^+^(59)	Inhibition of T cell response

The term “regulatory B cells” were firstly introduce by Bhan and Mizoguchi. Using T-cell receptor (TCR)-α^−/−^mice, μMT mice, and TCR-α^−/−^μMT mice, they found that colitis pathogenesis does not require B cells, but B cells are presumably involved in the elimination of apoptotic cells, which contributed to suppressing colitis (60). Similarly, Michael Hahne et al. reported that LPS-activated B cells expressing FAS ligands (FasL) can clear activated T cells such as FAS-expressing T cells, and transfer of LPS-activated B cells could ameliorate the development of diabetes in NOD mice (61). Subsequently, Atsushi Mizoguchi et al. found that under conditions of chronic enteritis, B cell subsets, characterized by upregulation of CD1d expression, can produce IL-10 and attenuate IL-1 upregulation and signal transducer and activator of transcription (STAT)3 activation, which indicates that B cells producing IL-10 could serve as regulatory cells in immunologically mediated inflammatory responses (34). Later, Claudia Mauri et al. used agonistic anti-CD40 and collagen to stimulate arthritic B cells, increasing the secretion of IL-10 in B cell subsets to control the proinflammatory Th1 type response while reducing secretion of interferon (IFN)-γ; the findings proved that these B cells play important roles in immune regulation in arthritis models (33). Niamh E. Mangan et al. have also reported that the induction of IL-10-producing B cells can modulate allergic responses in worm-infected mice (62, 63). Besides, studies of Bregs in transplantation have also been conducted. Lal Girdhari et al. proved that CD40 costimulatory blockade induces IL-10 producing Marginal Zone Precursor (MZP) Bregs, especially IL-21R^+^ MZP Bregs, performing a key function in restoring graft survival (50).

## MSCS Play Anti-Inflammatory Roles in Immune Diseases by Increasing Bregs

There have been many discoveries shown that MSCs exert immunomodulatory functions to affect B cells. In 2006, Anna Corcione et al. first discovered that hMSCs can directly interact with B cells to prevent their proliferation and death while promoting arrest during the G0-G1 phase of the cell cycle. They found that the expression of CXCR4, CXCR5, and CCR7 in B cells was downregulated as a result of inhibition of human B cell proliferation, differentiation into antibody-secreting cells, and chemotaxis *in vitro* (64). In 2007, Patrizia Comoli et al. reported MSCs induced by allo-stimulation *in vitro* are capable of modulating B-cell allo-responses via inhibiting antibody production, suggesting that third-party MSCs are able to suppress allo-specific antibody production *in vitro*, and may therefore help overcome a positive cross-match in sensitized transplant recipients (65). In 2009, it was reported that MSCs inhibit B cell terminal differentiation by releasing cytokines to downregulate B cell Blimp-1 expression both *in vitro* and *in vivo* (66). Moreover, Elisabetta Traggiai et al., through polyclonal stimulation of B cells isolated from children with systemic lupus erythematosus and healthy donors, found that bone marrow MSCs can promote the proliferation of transitional cells and naive B cells and their differentiation into immunoglobulin-secreting cells, moreover, MSCs strongly promote the proliferation of memory B cells and their differentiation into plasma cells (67).

Many previous studies have focused on MSCs inducing the production of regulatory T cells to exert immunosuppressive functions. Similarly, the modulation of regulatory B cells by MSCs also plays important roles in the treatment of many diseases. For example, in experimental autoimmune encephalomyelitis (EAE), an experimental model of human multiple sclerosis (MS), CD1d^high^CD5^+^ regulatory B cells were upregulated after MSC administration and exert anti-inflammatory and immunosuppressive effects (68). Experiments have also found that human umbilical cord MSCs (hUC-MSCs) protect experimental mice by increasing the numbers of CD5^+^ Bregs that produce IL-10 and correcting Treg/Th17/Th1 imbalance in a colitis model (69). Furthermore, Y Peng et al. have reported that the numbers of CD5^+^IL-10^+^ regulatory B cell subset are increased in patients with refractory chronic graft-versus-host disease after MSC treatment (70). Minglu Yan et al. reported that human synovial membrane-derived MSCs can inhibit the maturation and differentiation of B cells; induce CD21^high^CD23^high^ transitional 2 (T2) cells, CD23^low^CD21^high^ marginal zone (MZ) cells, and CD5^+^CD1d^+^IL-10 cells in the spleen; and increase the numbers of immature transitional B cells, such as IL-10^+^ cells, thus reducing the severity of arthritis in mice (71). Kunal S. Gupte et al. reported that co-culture of adipose tissue-derived MSCs (AD-MSCs) from 15 potential kidney donors with peripheral blood PBMCs could induce IL-10-secreting B cells, demonstrating the promise of cell therapies for immune diseases after transplantation (72). Studies demonstrated that MSC infusions contributed to long-term stabilization of renal allograft function, likely via triggering an active peripheral immunomodulation to induce long term immunophenotyping of naïve and CD24^high^CD38^high^ transitional B-cell subsets in kidney allograft recipients (73). Along with this, another recent study held by Davide Piloni el at. proved that CD19^+^CD24^high^CD38^high^ Breg cell subset also showed key functions in the long term acceptance of lung graft (74). These discoveries of B-cell subsets provide not only a potential marker of MSC-induced immunomodulation associated with transplantation tolerance, but also a prospective view in IL-10 producing B cells key functions among SOT applications. Recently, Di Lu et al. found that allogeneic MSC transplantation can promote the levels of IL-4 and IL-10 and the induction of Bregs in an aGVHD mouse model with complete mismatch of MHC and significantly inhibit the expression of CD69 and CD86 on B lymphocytes to prolong survival, thus demonstrating that B lymphocytes play an important role in the development of aGVHD and that B lymphocytes are targets of the immune regulatory cascade in MSCs (75). Studies based *in vitro* experiments or preclinical and clinical researches have reported the induction of Bregs by MSCs as we summarized in Table 2.

**Table 2:** Summary of the studies on MSC-mediated effects to Bregs.

**Study**	**Disease or study type**	**Key findings**
Chen et al. (76)	Clinical trial: BOS after allo-HSCT	Increased CD5^+^B cells and IL-10-producing CD19^+^CD5^+^ Bregs
Chen et al. (59)	Colitis model	Induced the novel CD23^+^CD43^+^Bregs subset
Planella et al. (77)	*Invitro* study	The PF as well as the CM could increase induced CD24^high^CD38^high^ B cells
Lu et al. (75)	Acute GVHD model	Decreased IL-4 and increased IL-10^+^Bregs
Li et al. (78)	EAE model	Increased CD5^+^ IL-10^+^ B cells
Mehdipour et al. (79)	*Invitro* study	Decreased TNF-α^+^/ IL-10^+^ B cells ratio in B cell-ASCs co-culture
Luk et al. (80)	*Invitro* study	Under immunological quiescent conditions, MSC increased IL-10^+^CD38^high^ CD24^high^ Bregs
Yan et al. (71)	CIA model	Increased CD21^high^CD23^high^ T2 cells, CD23^low^CD21^high^ MZ cells, and CD5^+^CD1d^+^IL-10^+^Bregs
Gupte et al. (72)	*Invitro* study	Increased IL-10-secreting Bregs from baseline of patients
Cho et al. (81)	Animal *in-vivo* study	Induced IL-10-expressing Bregs in an EBI3-dependent manner
Zhang et al. (82)	Clinical trial: NS after allo-HSCT	Induced CD19^+^CD5^+^IL-10^+^ Bregs
Hermankova et al. (83)	*Invitro* study	IFN-γ-treated MSCs inhibited IL-10 production by activated B cells via cell-contact and the Cox-2 pathway
Chao et al. (69)	Colitis model	Boosted the numbers of CD5^+^ B cells and IL-10-producing CD5^+^ Bregs
Peng et al. (70)	Clinical trial: refractory cGvHD	Increased IL-10-producing CD5^+^ B cells
Franquesa et al. (84)	*Invitro* study	Reduced plasmablast formation and induce IL-10-producing CD19^+^CD24^high^CD38^high^ Bregs
Park et al. (85)	SLE model	Increased IL-10-producing Bregs
Garimella et al. (86)	CIA model	Increased the CD19^+^CD1d^high^CD5^+^ Bregs in the spleens of ASC-treated CIA mice
Wang et al. (87)	Cardiac allograft model	MSC-expressing B7-H1 neutralization reduced IL-4^high^IL-10^high^CD83^low^ B cells
Guo et al. (68)	EAE model	Upregulated CD1d^high^CD5^+^Bregs

*BOS, Bronchiolitis obliterans syndrome; HSCT, Hematopoietic Stem Cell Transplantation; GVHD, graft-versus-host disease; EAE, experimental autoimmune encephalomyelitis; CIA, collagen-induced arthritis; NS, Nephrotic syndrome; SLE, systemic lupus erythematosus*.

## How MSC_S_ Regulate Breg Generation

Accumulating evidence has revealed the importance of Bregs and Tregs in the maintenance of immune tolerance, and MSC-mediate disease improvements are often associated with the induction of Bregs and Tregs. It's well-known that MSCs regulate Tregs proliferation, survival, and function mainly through several pathways. Firstly, cell-to-cell contact, through which interactions among different molecules expressed by MSCs and T lymphocytes (such as ICOSL and ICOS, Notch and Notch ligands), upregulates the production of IL-10 and the proliferation of Tregs. Followed, the secretion of soluble factors by MSCs, including TGFβ, CCL2, IL-6, IL-7, PGE2, IDO, HO-1, and HLA-G5, can regulate Treg generation (88–90). Moreover, antigen-presenting cell dependence; in this pathway, MSCs affect antigen-presenting cells (dendritic cells, monocytes, macrophages) to induce regulatory phenotypes and promote Treg activity through IL-10 and TGF-β1, although the detailed mechanism has not been fully elucidated. In addition, MSC-derived extracellular vesicles, containing specific RNA, proteins and other biological molecules, induce the polarization of CD4^+^ T cell into Tregs (91).

**Figure 1:**
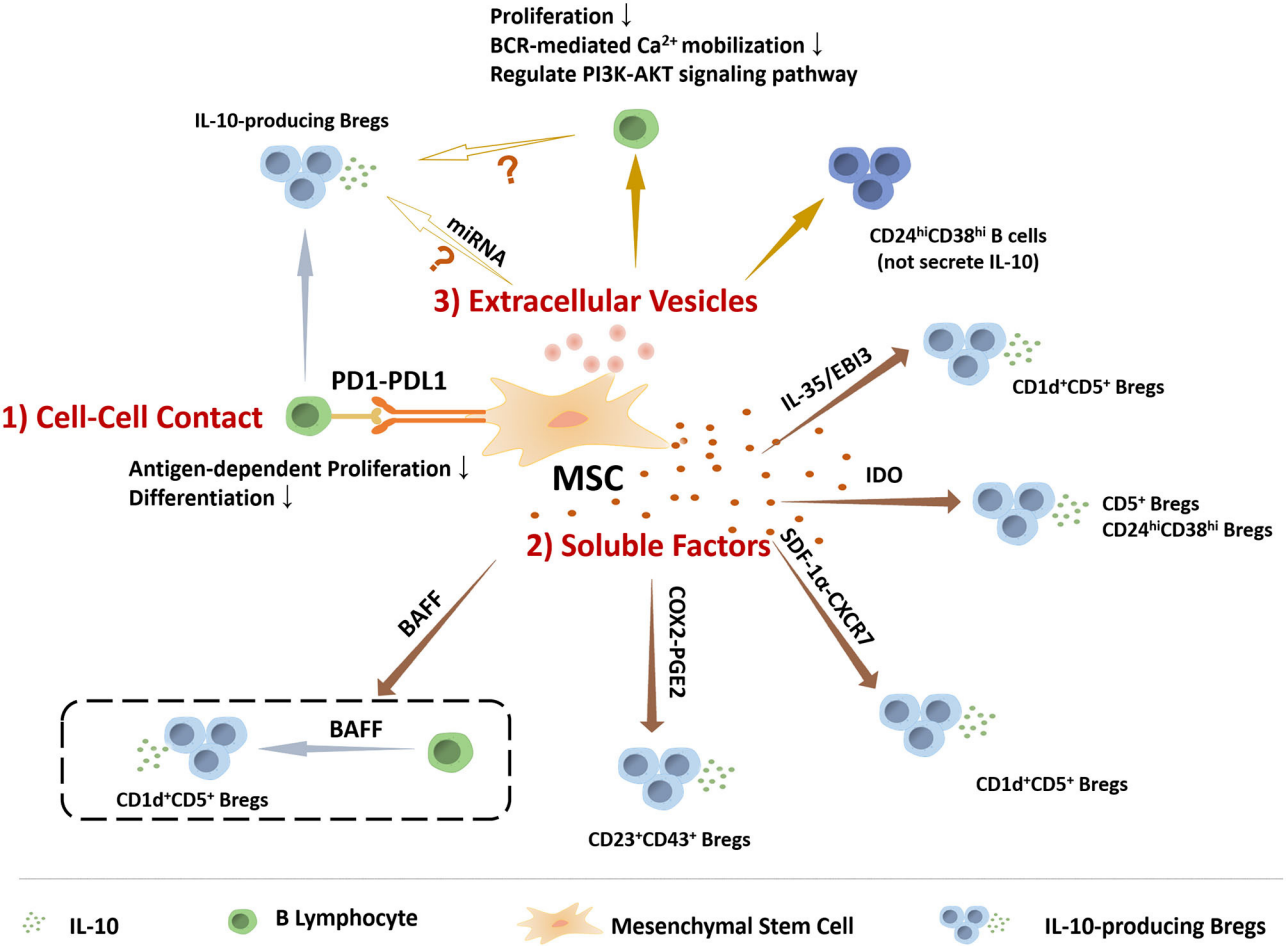
The role of MSCs in regulating the IL-10 producing regulatory B cells. MSCs perform functions on modulating IL-10 producing regulatory B cells via many manners, including (1) Cell-to-cell contact: MSCs play roles in B cells via PD1-PDL1 pathway to inhibit antigen-dependent proliferation and differentiation, and induce Bregs. (2) Soluble factors: IL-10-producing Breg subsets, including CD5^+^ Bregs, CD24^high^CD38^high^ Bregs, CD1d^+^CD5^+^ Bregs, and CD23^+^CD43^+^ Bregs, are mediated by MSCs-secreting soluble factors. (3) Extracellular Vesicles: MSCs-EVs could inhibit B cell proliferation and BCR-mediated Ca^2+^ mobilization, regulate PI3K-AKT signaling pathway in B cells that is critical for Breg cell development, and induce CD24^high^CD38^high^ B cell subpopulation, a classic phenotype of Bregs, but without IL-10 production. Based on the current data, MSCs-EVs might be involved in MSCs regulating IL-10 producing B cells.

Compared to MSCs inducing Tregs, the specific mechanisms by which MSCs regulate the generation of Breg are still not sufficiently clear. Several studies have focus on the mechanism that induces the generation of Bregs. H Li et al. found that T follicular regulatory (Tfr) cells could induce IL-10^+^ Breg cells, as higher frequency of IL-10^+^ Breg cells was observed when incubation with Tfr cells (92). Moreover, tolerogenic DC (tolDC), one type of DC with immuno-suppressive properties, were reported to induce the IL-10 producing Breg, as wells as the IL-10 producing type 1 regulatory T cells (Tr1) (93). Cynthia M. Fehres et al. described that a proliferation-inducing ligand (APRIL) induced IL-10^+^B cells production in EAE and CHS models, as APRIL promoted the differentiation of naïve human B cells to IL-10-producing IgA^+^ B cells (94). It has been postulated by some investigators that the conditions in the microenvironment are key factors for the induction of Bregs. Notably, Toll-like receptor (TLR), CD40, and BCR-induced signaling are vital for Breg function (95–97). In view of previous studies that have assessed multiple modulatory mechanisms of MSCs, we illustrate below the relationship between MSCs and Bregs from several perspectives, which also summarized in the Figure 1.

### Cell-to-Cell Contact

MSCs can regulate immune responses through direct cell-to-cell contact. Via interaction with surface molecules and/or receptors, MSCs might directly regulate their downstream pathways in B cells, thereby affecting B cell activation, proliferation, survival, differentiation, and Bregs induction. For instance, M. Franquesa et al. experimentally demonstrated that hASCs can act independently of T cells and directly on B cells to promote the production of CD19^+^CD24^high^CD38^high^ and IL-10-production regulatory B cells (84). Although cell-to-cell contact manner have been confirmed to be involved in MSCs inducing Bregs by the transwell co-culture (59, 80), little is known about the particular molecules. One of the major molecules involved in this cell-to-cell interaction of MSCs is the costimulatory molecules is programmed death ligand-1 (PD-L1, also known as B7-H1). PD-L1 is well-known for its role in immune checkpoint regulation (98). Its receptor, programmed cell death protein 1 (PDCD1; also known as PD1), is an immunoglobulin-superfamily member that over-expressed upon programmed cell death as its primary function described to attenuate the immune response (99, 100). Francesca Schena et al. found that BM-MSCs inhibit antigen-dependent proliferation and differentiation of follicle and MZ B cells *in vitro* through the PD-1/PD-L1pathway, and ameliorate the inflammatory response in systemic lupus erythematosus mice (101). H Wang et al. reported that the expression of B7-H1 on MSCs was required for IL-10-producing Bregs development in recipients and MSC-mediated suppression of antibody production and B cell proliferation, which contribute to the induction of immune tolerance to allografts in mouse cardiac allograft model by the combination therapy of MSCs and rapamycin (RAPA) (87).

### Soluble Molecule Interactions

#### Cyclooxygenase-2 (COX-2)/PGE2

Prostaglandins (PGs) are small molecule derivatives of arachidonic acid produced by cyclooxygenase (102). Prostaglandin E2 (PGE2), the main product of cyclooxygenase in myeloid cells and stromal cells, is a biologically active factor whose synthesis was regulated by COX-2 and shown to regulate multiple aspects of inflammation in immune cells (103). Many studies have shown that MSCs exert their therapeutic ability mainly dependent on PGE2 secretion (104, 105). MSCs-derived PGE2 also contribute to their induction of Tregs (106). In B cells, Tae-Hoon Shin et al. show that COX-2 signals are necessary for MSCs to inhibit the proliferation and maturation of B lymphocytes, result of inhibiting the secretion of IgE by mature B cells in a mouse atopic dermatitis (AD) model (107). R Chen et al. shown that PGE2 could induce B10 cells via the MAPKs/AKT-AP1 axis or aryl hydrocarbon receptor (AhR) signaling (108). Recently, COX-2/PGE2 pathway is also found to involved in MSCs induce CD23^+^CD43^+^ Bregs, which significantly reducing the clinical and histopathological severity of induced colon inflammation and ameliorating gastrointestinal mucosal tissue damage in mice (59). However, IFN-γ-primed MSCs were reported to inhibit the production of IL-10 by LPS-activated B cells through the COX-2 pathway (83). PGE2 has shown to exert paradoxes function in regulating immune response (103), more experiments might need to uncover the key mechanisms and targets of PGE2-mediated effects on MSCs inducing Bregs. The immune status of MSCs may be another cause needed pay attention to, as the microenvironment is one of the major factors that affecting the immuno-regulatory ability of MSCs.

#### Indolamine-2,3-dioxygenase (IDO)

IDO catalyzes the first and rate-limiting step of tryptophan catabolism in the kynurenine pathway, and its downstream metabolites include kynurenine (KYN) and 3-hydroxyanthranilic acid. It is worth noting that IDO has been shown to regulate the expression of inflammation-related genes, either by itself as a signaling factor or through the production of biologically active intermediates via the kynurenine pathway, such as 3-hydroxyanthranilic acid and kynurenic acid (KYNA). IDO could inhibited T cell proliferation and modulated regulatory T cell differentiation (109, 110). G Wang et al. demonstrated IDO is necessary to the therapeutic effects of human umbilical cord-derived MSC (hUC-MSC) for treating acute lung injury (ALI) (111). Based on previous studies, the IDO expression in MSCs require priming by IFN-γ and pro-inflammatory cytokines that enhance IDO levels via JAK/STAT signaling (112, 113). IFN-γ-pretreated MSCs inhibit the production of IgG and the proliferation of B cells, largely dependent on tryptophan catabolism by IDO (80). Human umbilical cord-derived MSCs (hUC-MSCs) can control EAE by increasing the proportion and promoting the function of CD5^+^IL-10^+^ B cells. After co-culture with MSCs, CD5^+^ B cells show a stronger ability to inhibit T cell proliferation and proimmflamatory cytokines secretion, as well as to induce Tregs (78), and these enhanced immunomodulation of CD5^+^ B cells by MSCs were reversed when blocking the IDO pathway. Moreover, MSCs increased the frequency of CD5^+^ Breg cells by enhancing their proliferation and survival via the IDO pathway (70).

#### IL-35

Interleukin-35 is a novel anti-inflammatory cytokine belonging to the IL-12 cytokine family that can be applied as a potential therapy for chronic inflammation and autoimmune diseases (114). Human IL-35, which functions as an important immuno-modulator, seems to inhibit mature inflammation rather than prevent inflammation as IL-35 is not constitutively expressed in human tissue (27, 115, 116). IL-35 has reported to induce both Tregs and Bregs (117). IL-35 could induce the conversion of B cells into Bregs, including IL-35^+^ Bregs and IL-10^+^ Bregs. Mice deficient in p35 or EBI3, the two subunits of IL-35, exhibit an exacerbation in EAE and experimental autoimmune uveitis (EAU) with less Bregs (27, 43). Studies have revealed that overexpression of IL-35 in hMSCs can increase the proportion of Tregs among lamina propria lymphocytes (LPLs) and induce an immunosuppressive microenvironment via inhibition of the expression of TNF-α, IFN-γ, and IL-17 in the lamina propria (114). Similarly, IL-35 also takes part in MSCs inducing Bregs. Kyung-Ah Cho et al. have proven that MSCs are capable of ameliorating B-cell activation induced by hormonal stimulation, and directly inducing the population of immunosuppressive IL-10-secreting Breg cells in an IL-35-dependent manner without acting on T cells; both these MSCs-mediated effects require MSCs-derived EBI3, a critical subunit of IL-35 (81).

#### SDF-1α-CXCR7

Stromal cell-derived factor-1α (SDF-1α, also known as CXCL12) is a crucial process involved in the chemotaxis of stem cells/progenitor cells (118). Previous studies have reported that the migration and survival of MSCs have been enhanced via up-regulation of SDF-1 receptors, CXCR4 and CXCR7, under hypoxic preconditioning stimulation, which likely contribute to improving the therapeutic effect in renal ischemia/reperfusion (I/R) injury in animal model (119). According to Marie-Luise Humpert et al. research, CXCR7, is an atypical chemokine receptor, binds CXCL12 and CXCL11 to regulate CXCR4/SDF-1-mediated the migration of plasmablasts during B-cell maturation (120). Moreover, Yan Qin et al. demonstrated that low concentration of SDF-1 promoted MSCs to induce IL-10-producing Bregs while high concentrated inhibited MSCs induction of IL-10^+^Breg cells, but overexpressed CXCR7 of MSCs can reverse this inhibitory effect. The result supported that SDF-1α-CXCR7 axis play key roles in MSCs regulating IL-10-producing Bregs, especially CD1d^+^CD5^+^IL-10^+^Bregs, by regulating paracrine actions (121). In addition, endometrial regenerative cells (ERCs), mesenchymal-like stromal cells, have been found to induce a donor-specific allograft tolerance in mouse cardiac allograft models, which is depended on SDF-1 mediated increasing levels of regulatory immune cells including IL-10 producing CD1d^high^ CD5^high^ CD83^low^ Bregs (122).

#### B Cell-Activating Factor (BAFF)

B cell-activating factor (BAFF) is a member of the tumor necrosis factor superfamily known to play a critical role in the survival and maturation of B cells by binding to the receptors BCMA (B cell maturation antigen) and TACI (transmembrane activator and CAML interactor) (123). BAFF is also critical for naive circulating B cell and MZ B cell homeostasis. BAFF is expressed in a wide variety of cell types, including macrophages, dendritic cells and neutrophils, and even functions in an autocrine manner (124). Using BAFF-transgenic (Tg) mice, BAFF has been demonstrated to induce CD4^+^Foxp3^+^ Treg cells to suppress T-cell responses (125), suggesting a regulatory role of BAFF *in vivo*. Followed, low dosages of BAFF was found to possess the ability to induce IL-10 producing Bregs with the phenotype of CD1d^hi^CD5^+^, moreover, the number of IL-10-producing B cells in the marginal zone regions were increased when treated with BAFF *in vivo* (126). Interestingly, MSCs were reported to express BAFF both in mRNA and protein (127), indicating that MSCs might have the ability to induce Bregs via secreting BAFF. In clinical studies, MSCs are shown to decrease the plasma levels of BAFF in patients with cGVHD or refractory rheumatoid arthritis (RA), accompanied with regulating the activity of B cells and alteration in B cell subpopulation (128, 129). However, more experiments still need to confirm the BAFF-mediated effects on MSCs inducing Bregs, and reveal the underling mechanisms.

### MSC-EVs

An increasing number of studies have shown that MSCs perform many paracrine functions by releasing extracellular vesicles (EVs). In particular, small EVs (50–200 nm in diameter) (130) can be obtained from cell culture supernatants of MSCs cultured under different culture conditions and have been reported to possess therapeutic effects in different preclinical models. MSC-derived exosomes function through horizontal transfer of proteins, mRNA, and regulatory microRNAs (131). MSC-EVs have become promising therapeutic agents (132). Drirh Khare et al. identified 39 upregulated genes by sequencing exosomes derived from MSCs cocultured with B cells, including SerpinB2, PTGS2, CXCL8 (IL8), and MZB1 (marginal zone B and B1 cell specific protein) (133–136). These genes are involved in a variety of classic immunosuppressive effects, including inhibition of T cell activation, B cell proliferation, and BCR-mediated Ca^2+^ mobilization, proving that mesenchymal stromal cell exosomes affect the expression and function of B lymphocytes (137). Recently, L Guo et al. reported that MSC-EVs prevent fibrosis of skin in sclerodermatous cGVHD mouse model via blocking the TFH/GC B cells interaction and reduce the ratio of BAFF to B cells *in vivo* (138). MSC-derived soluble protein-enriched fractions (MSC-PFs) have effects comparable to those of MSCs and can promote B cells to produce IL-10. MSC-EVs induce CD24^high^CD38^high^ B cells to the same extent as MSCs while the resulting cells do not produce IL-10 (77). MiR-155, a microRNA that significant increase in MSCs prime with IFN-γ and TNF-α (139), promotes IL-10 production in CD24^hi^CD27^+^ Bregs directly by inhibiting the expression of Jarid2, resulting in reduction of H3K27me3 binding to the IL10 promoter (139). In addition, MSCs-EVs are found to regulate the PI3K-AKT signaling pathway in B cells (140), combined with PI3K-Akt pathway in B cells is critical for Breg cell development, it is conceivable that MSCs might regulate the Bregs via their EVs to modulate the PI3K-AKT signaling pathway in B cells. Of course, there are still many unknowns in this field, and more research is needed to uncover the role of MSCs-EV in regulating Bregs.

Nevertheless, the potential for MSC-EVs immunomodulation remains promising, although the mechanisms of MSC-EVs in Breg induction is not yet well-understood. Moreover, MSC-EVs are traditionally derived from highly heterogeneous MSC cells. Due to the diversity of MSCs, the complexity of MSCs preparation, the lack of standardized quality assurance procedures for various methods of production and isolation of EVs, and the limited reproducibility of *in vitro* and *in vivo* functional assays. Four associations (SOCRATES, ISEV, ISCT, and ISBT) have proposed specific harmonized standards for MSC-EV preparation, which will help promote the development of clinical applications in this field (141).

## Conclusions

Investigations in the past few years have provided new insights into the functions of MSCs in immune system modulation and the potential of MSC-based cell therapies, which have been extensively assessed in clinical studies for their efficacy in degenerative, autoimmune, or inflammatory diseases. The mechanisms by which MSCs perform their therapeutic functions are multifaceted, but in general, these cells are thought to be able to balance the inflammatory and regenerative microenvironment of damaged tissue in the presence of severe inflammation. Studies on the interactions between immune systems and MSCs have shown that enhancement of the immunoregulatory activity of MSCs is essential during tissue regeneration. Over past decades, numerous studies have been conducted to clarify the immunomodulatory effects of MSCs on immune cells. Completed and ongoing clinical trials and *in vivo* studies on the therapeutic effects of MSCs against immune-mediated diseases have proven that MSCs can increase the generation of Bregs. It has been suggested that MSCs can increase the secretion of IL-10 by Bregs to treat inflammatory diseases, but research on specific mechanisms is still relatively scarce. Undeniably, the effectiveness of related B cell-based treatments greatly depends on the functions of Bregs, especially IL-10-secreting Bregs. Numerous studies on Bregs have revealed that B10 cells have powerful potential to ameliorate inflammatory disorders, exhibiting promise for use in the treatment of autoimmune diseases. On the one hand, regulatory B cells have not been clearly defined, and there is a lack of identified markers. At present, Bregs are still defined on the basis of their functions, which make breakthroughs in related research difficult. We have reviewed previous studies on effective MSC-mediated promotion of the production of IL-10^+^ Bregs. To a certain extent, MSCs have multiplicative potential; they are able to induce Bregs and/or increase Breg production through a wide range of verified direct and indirect mechanisms. In the future, further studies are needed to discover reliable markers for defining different subpopulations of Bregs, clarify the heterogeneity among different subpopulations of Bregs used in specific treatments and clarify the potential mechanisms by which MSCs regulate Bregs. In clinical applications of MSCs combined with Bregs for the treatment of immune diseases, the stability and flexibility of the treatments should be closely considered and optimized to achieve appropriate modulation of inflammatory responses at different stages of disease progression.

## Author Contributions

JL and XC searched the literature and wrote the manuscript. XC and QL critically revised the manuscript and final approval of the work. All authors contributed to the article and approved the submitted version.

## Conflict of Interest

The authors declare that the research was conducted in the absence of any commercial or financial relationships that could be construed as a potential conflict of interest.
